# A phloem‐based defense mechanism linked to elevated riboflavin levels in wild tomato *Solanum chmielewskii* impedes whitefly nymphal development

**DOI:** 10.1111/tpj.70363

**Published:** 2025-07-25

**Authors:** Lissy‐Anne M. Denkers, Arjen van Doorn, Marc Galland, Gerd Balcke, Martin de Vos, Robert C. Schuurink, Petra M. Bleeker

**Affiliations:** ^1^ Department of Plant Physiology, Green Life Science Research Theme, Swammerdam Institute for Life Sciences University of Amsterdam Amsterdam The Netherlands; ^2^ KeyGene N.V. Wageningen 6708 PW The Netherlands; ^3^ Department of Cell and Metabolic Biology Leibniz Institute of Plant Biochemistry Weinberg 3 Halle (Saale) 06120 Germany; ^4^ Present address: Roche Diagnostics GmbH Munchen Germany; ^5^ Present address: INRAE, Institute of Genetics, Environment and Plant Protection (IGEPP—Joint Research Unit 1349) Le Rheu France; ^6^ Present address: KWS Vegetables BV Wageningen The Netherlands

**Keywords:** *Bemisia tabaci* (silverleaf whitefly), grafting, nymph development, phloem, Random Forest, riboflavin, *Solanum chmielewskii*, *Solanum lycopersicum* (tomato)

## Abstract

Management of the phloem‐feeding pest insect *Bemisia tabaci* (whitefly) is difficult due to its short generation time and large number of offspring. Several whitefly‐resistant wild tomato accessions have been identified, with the resistance attributed to specific defense metabolites in glandular trichomes. Interestingly, we found that on *Solanum chmielewskii* LA1840, which lacks trichome‐based resistance, nymphal development is delayed and decreased compared with a cultivated tomato. Here, we show that the resistance observed in LA1840 is based on a mobile factor in the vasculature, the site of interaction for nymphs during feeding. The putative compound responsible for the resistance apparently passed the graft junction from an LA1840 rootstock to an otherwise susceptible cultivar scion. After untargeted metabolomics on the phloem collected from the wild accessions, a Random Forest algorithm predicted riboflavin to be linked to the resistance phenotype. The resistant genotypes indeed exhibit increased riboflavin levels in leaves compared with susceptible plants. The effect of elevated riboflavin levels on whitefly nymph development was validated through feeding riboflavin to susceptible plants. Our results highlight the power of natural variation in metabolites and vasculature‐based resistance mechanisms for the development of sustainable whitefly management.

## Significance Statement

Wild tomato *Solanum chmielewskii* harbors a resistance against the herbivorous pest insect *Bemisia tabaci* (whitefly) that was linked to elevated concentrations of riboflavin in this tomato species. The mechanism is phloem transportable and causes hampered development of the whitefly nymphs feeding on the plant.

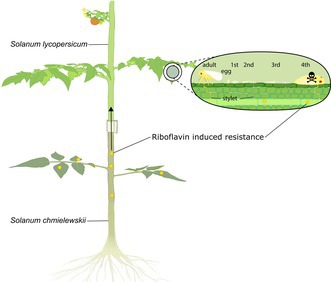

## INTRODUCTION

Phloem‐feeding insects, such as the whitefly *Bemisia tabaci*, are major pests in agriculture and cause massive yield losses (Gusmao et al., 2006). Due to their stealthy feeding style, during which they penetrate the phloem with their stylet without causing considerable cell damage, they bypass most of the plants' defense mechanisms (Walling, 2008). During feeding, whiteflies inject saliva into the phloem, which has been shown to contain, among other things, RNAs and proteins that can act as salivary effectors which function in suppressing the plant immune response (Lee et al., 2018; Su et al., 2015; van Kleeff et al., 2016). A direct effect of a high‐density whitefly infestation can be nutrient depletion. Moreover, indirectly, whiteflies can be extremely harmful in their role as viral‐vector insects. With their probing and feeding behavior, *B. tabaci* can transmit a multitude of different viruses that pose a significant threat to agriculture worldwide, including well‐known begomoviruses, such as tomato yellow leaf curl virus (Jones, 2003; Navas‐Castillo et al., 2011; Polston et al., 2014).

*B. tabaci* is a polyphagous insect, and as such, has an extremely broad host range including many economically important plant species. Hence, whitefly infestations and consequent outbreaks of viral diseases are a common occurrence in tomato (*Solanum lycopersicum*). Resistance to whitefly has been found for several wild tomato relatives (Bleeker et al., 2011; Lucatti et al., 2013; McDaniel et al., 2016). The most studied defense mechanism against insects in wild tomato species thus far is the production of specialized metabolites in glandular trichomes. Trichomes are hair‐like epidermal structures which have been categorized as either glandular or non‐glandular types (Glas et al., 2012). Particularly, trichomes accumulating acylsugars and sesquiterpenes have been reported to exhibit bioactivity against *B. tabaci* (Kortbeek et al., 2021; Marchant et al., 2020; Rakha et al., 2017; Vosman et al., 2019). However, not all wild tomato species are resistant to whiteflies. In an adult whitefly survival assay, the wild accession *S. chmielewskii* LA1840 scored comparably to a susceptible cultivar (Kortbeek et al., 2021). Still, whitefly oviposition and nymph development were found to be hampered on two *S. chmielewskii* accessions, including LA1840 (de Almeida et al., 2023). This indicates that this wild tomato species harbors a defense mechanism other than surface chemistry, which we hypothesized to be localized in the phloem. A phloem‐based resistance mechanism could be expected to target the nymphs rather than the adult survival since three of the four nymphal stages are sessile and feed continuously from the phloem (Stansly et al., 2010). Adults, on the other hand, only feed occasionally and may survive on food with a lower quality than needed by the nymphs to develop.

Although to the best of our knowledge no phloem‐based resistance mechanisms have been identified in wild tomato so far, previous findings do suggest such mechanisms exist. For example, the population densities of *B. tabaci* and the greenhouse whitefly *Trialeurodes vaporarioum* in free‐choice bioassays were lower on a susceptible tomato cultivar when the scion was grafted onto resistant rootstock cultivars (Mandušić et al., 2019; Žanić et al., 2017). Although the exact mode of action of these resistances remained unknown, these findings suggest that resistance from a rootstock can be transported to the scion and might therefore be phloem‐based. In other plant species, several phloem‐based resistance mechanisms have been identified, including the blocking of sieve plates upon damage on sieve tube (as reviewed by Jiang et al., 2019 and Twayana et al., 2022). Furthermore, in the phloem of a wide array of plant species, specialized metabolites linked to insect resistance have been found, including alkaloids (Dreyer et al., 1985; Lee et al., 2007; Wink et al., 1982; Wink & Witte, 1984), glucosides (Gowan et al., 1995; Lohaus & Schwerdtfeger, 2014; Malka et al., 2016; Merritt, 1996) and terpenes (Wallis et al., 2008; Yu et al., 2020).

Considering the above, we hypothesized that *S. chmielewskii* might harbor a phloem‐based resistance mechanism. To test this, we followed the development of whitefly nymphs on four different *S. chmielewskii* accessions, including LA1840, a susceptible cultivar, and grafts made of LA1840 and the cultivar. Using an untargeted metabolomics approach followed by a Random Forest machine learning feature selection method, we predicted features with a significant importance to the observed phenotype. One of these features was identified as riboflavin, a compound present in higher concentrations in the resistant *S. chmielewskii* accessions and in an introgression line originating from a cross between LA1840 and a tomato cultivar. The effect of riboflavin levels on nymphal development was validated in experiments where the compound was either fed to leaves of the susceptible cultivar or its biosynthesis inhibited in LA1840, using a hydroponics setup.

## RESULTS

### A hampered whitefly development phenotype is observed in some *S. chmielewskii* accessions

**Figure 1:**
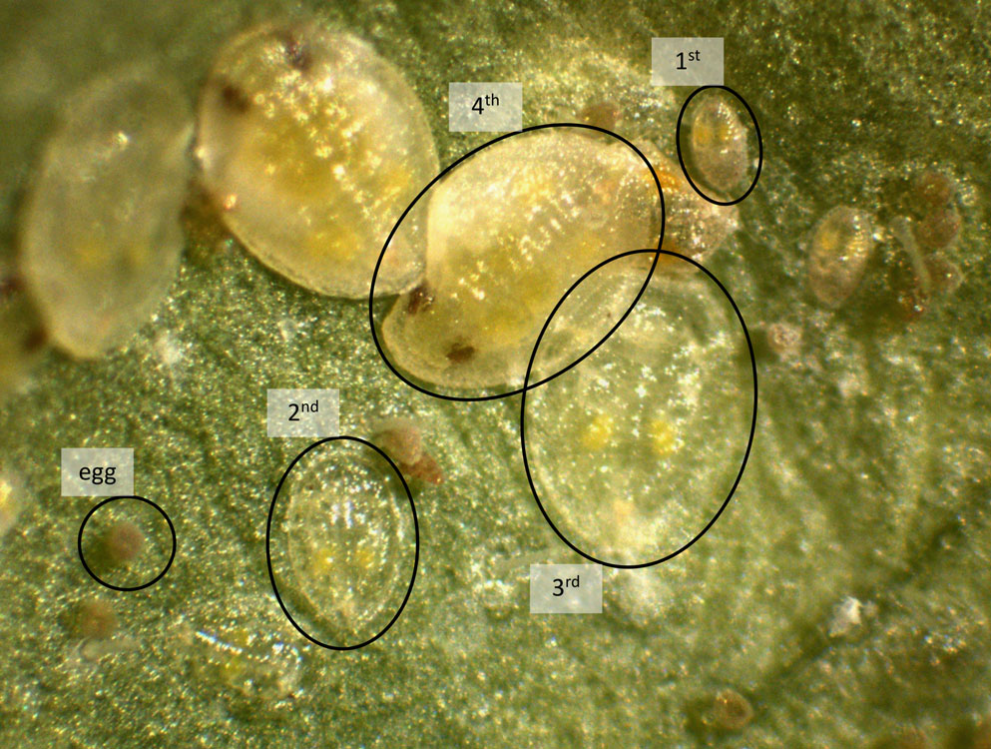
Photograph exemplifying the different whitefly (*Bemisia tabaci*) developmental stages on a cucumber leaf. Indicated are eggs and first (mobile stage), second, third, and fourth (immobile stages) instar nymphs of *B. tabaci* (MEAM1) used in this study.

**Figure 2:**
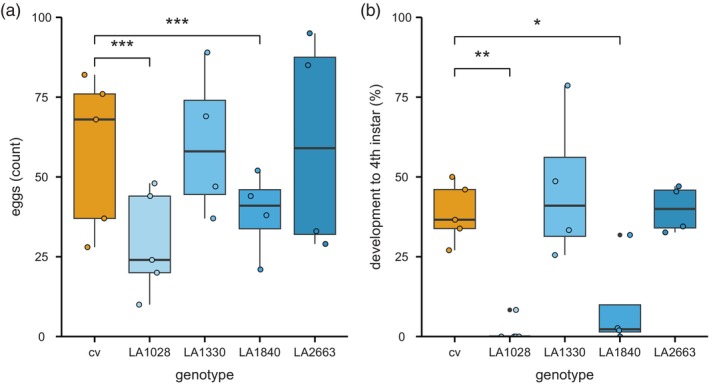
Whitefly resistance phenotype in *Solanum chmielewskii* accessions LA1028, LA1330, LA1840, and LA2663 (blue), compared with tomato cultivar *S. lycopersicum* cv Moneymaker (cv; orange). (a) Whitefly oviposition as number of eggs deposited by 10 female whiteflies after 3 days on four wild accessions and cv (GLM: df = 21, AIC = 316.5). (b) Percentage of eggs that develop to fourth instar after 24 days on the four wild accessions compared with cv [Kruskal–Wallis: *χ* ^2^(4) = 14.80, *P* = 0.005]. Boxes represent the interquartile range (IQR), displaying the median as bold line (cv and LA1028: *n* = 5; LA1330, LA1840, and LA2663: *n* = 4), whiskers 1.5 × IQR. Colored dots represent individual datapoints, black dots are outliers. Asterisks indicate *P*‐values from the comparison of LA1028, LA1330, LA1840, and LA2663 to cv using a generalized linear model or a Kruskal–Wallis rank sum test followed by a non‐parametric multiple comparison for relative effects (***P < 0.001; **P < 0.01; *P < 0.05).

To investigate whether *S. chmielewskii* exhibits an increased resistance to whitefly, we performed a no‐choice whitefly developmental bioassay on four *S. chmielewskii* genotypes (LA1028, LA1330, LA1840, and LA2663) which we confirmed did not differ in leaf surface chemistry (Figure S1) and used the susceptible cultivar *S. lycopersicum* Moneymaker (cv) as a control. Ten female whiteflies were allowed to deposit eggs for 72 h on one leaflet of the fourth, fully developed leaf from the top. After *B. tabaci* eggs hatch, the nymphs go through four nymphal stages (instars) before reaching their adult stage (Figure 1). First, the number of eggs laid on different accessions was counted, and during 24 days, the number of nymphs that reached the fourth instar stage was determined. There was a reduced oviposition rate on LA1028 and LA1840 compared with the cultivar (Figure 2a; Table 1), and the host plant genotype also affected development from eggs to the fourth and final instar stage (Figure 2b; Table 1). On the same two accessions, LA1028 and LA1840, nymphal development was clearly hampered compared with the cultivar and two other *S. chmielewskii* accessions, indicating natural variation in a resistance mechanism that specifically seems to target the whitefly nymph development.

**Table 1:** Number of eggs, number of fourth instars, and development as a percentage of eggs developed to fourth instars (*n* = 5) reared on leaves of *S. lycopersicum* cv Moneymaker (cv) and *S. chmielewskii* accessions LA1028, LA1330, LA1840, and LA2663

Genotype	Eggs	Fourth instars	Development to fourth instars
	Mean (±SE)	Mean (±SE)	Mean (±SE)
cv	58.20 (±10.82)	22.40 (±4.70)	38.70% (±4.16)
LA1028	29.20 (±7.26)	0.40 (±0.40)	1.67% (±1.67)
LA1330	60.50 (±11.62)	30.75 (±13.28)	46.54% (±11.73)
LA1840	38.75 (±6.57)	4.00 (±3.34)	9.09% (±7.60)
LA2663	60.50 (±17.17)	24.00 (±6.96)	39.91% (±3.70)

Values are given as mean with the standard error (SE) in parentheses.

**Figure 3:**
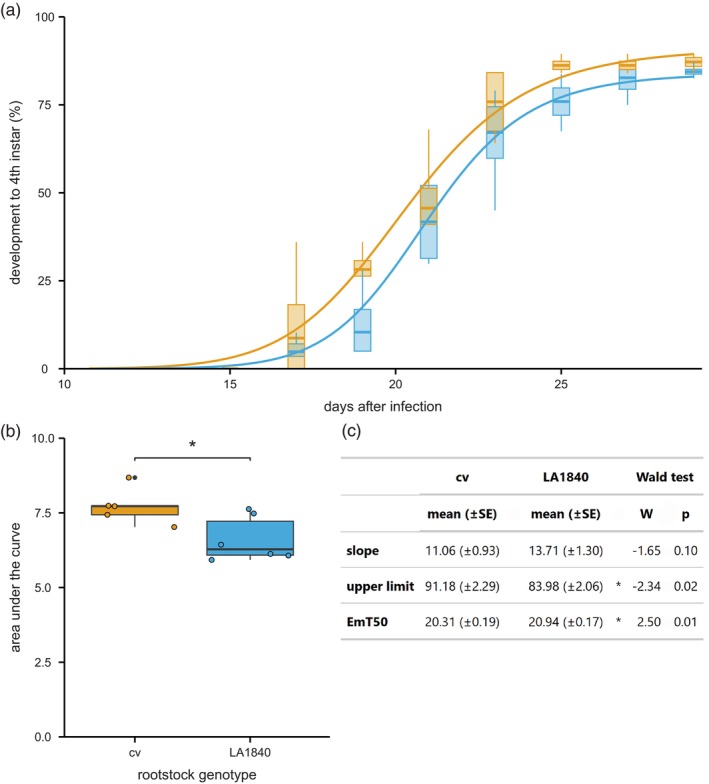
Whitefly resistance phenotype in *S. lycopersicum* cv Moneymaker scions grafted on *S. chmielewskii* LA1840 rootstocks. (a) Three‐parameter log‐logistic models (curves) fitted to the observed development of hatched eggs to fourth instar stage over time (boxes) on Moneymaker scions grafted on *S. lycopersicum* Moneymaker rootstocks (cv; orange) compared with Moneymaker scions grafted on *S. chmielewskii* LA1840 (blue). Days after infection indicated on *x*‐axis start at 10. (b) Area under the curve for the observed development from first to fourth instar per clip cage as proportion on LA1840 grafts (6.61 ± 0.31 SE) compared with cv grafts (7.72 ± 0.27 SE; *t*‐test, *t*_9.00_ = 2.71, *P* = 0.024). Boxes represent the interquartile range (IQR), displaying the median as a bold line (cv: *n* = 5; LA1840: *n* = 6), whiskers 1.5 × IQR. Colored dots represent individual datapoints, black dots are outliers. The means were compared using a two‐sample *t*‐test, as indicated with asterisks (*P < 0.05). (c) Slope, upper limit, and 50% emergence time in days (EmT50) corresponding to the parameters of the log‐logistic models in a, compared using a Wald test. Values are given as mean with the standard error (SE) in parentheses. *P < 0.05.

As nymphs become immobile after the 1st instar stage and remain attached to the plant phloem, continuously obtaining nutrients, we decided to test if the observed resistance is due to a phloem‐based, mobile factor. To this end, we grafted cultivar scions onto either LA1840‐ or cultivar control rootstocks. At grafting, the rootstocks had one or two true leaves and axillary meristems still attached. We decided to use LA1840 as a resistant genotype instead of LA1028 because a collection of introgression lines (ILs) originating from a cross between *S. lycopersicum* cv Moneyberg (cv) and *S. chmielewskii* LA1840 was available to study the phenotype in more detail (Ballester et al., 2016). Next, a whitefly development bioassay was performed on the cv scions, comparing the effect of grafting onto the wild tomato accession and a control cultivar rootstock. Albeit less extreme compared with the observations on the original resistant accession LA1840, the resistance phenotype of hampered nymph development was mirrored on the cv|LA1840 grafts (graft annotation: scion|rootstock; Figure 3). Although the number of eggs per clip cage appeared slightly higher on cv|LA1840 grafts compared with cv|cv grafts, the final egg hatching rate was similar for cv|LA1840 and cv|cv grafts (Figure S2; Table S1).

After hatching, we closely followed the development through all four nymphal stages and fitted a log‐logistic mathematical model on the development over time (Figure 3a). The effect of the rootstock genotype increased with each developmental stage and was strongest at the fourth instar stage. We therefore used the development from first to fourth instar stage to compare the effect of the vasculature connection to the two rootstock genotypes. The log‐logistic model for cv|cv grafts had an upper limit of 91.18 (±2.29 SE), indicating an average maximum of 91.18% of first instar nymphs on cv grafts developing to fourth instar stage. For the cv|LA1840 grafts, the model only reached an upper limit of 83.98 (±2.06 SE). Besides this, the developmental speed from first to fourth instar was also higher on cv|cv grafts than on cv|LA1840 grafts. The cv|cv model reached the EmT50 (time at which 50% of first instar nymphs have developed to fourth instar) at 20.31 (±0.19 SE) days, while the cv|LA1840 model reached EmT50 at 20.94 (±0.17 SE) days (Figure 3c). Taken together, although the effect was weaker when compared with performing the assay on a full wild accession, the development to fourth instar stage on grafted plants was slower and fewer nymphs reached fourth instar stage on the cv|LA1840 grafts compared with the same cv grafted onto a cv control rootstock.

Finally, to compare the developmental speed and maximum percentage of fully developed nymphs on the cv|cv and cv|LA1840 grafts, we calculated the area under the developmental curve for the individual replicates. Doing so, we obtained a variable that could be compared statistically between treatments (Figure 3b). The area under the curve was higher for cv|cv grafts than for cv|LA1840 grafts, indicating a hampered nymphal development due to the LA1840 rootstock. Because whitefly nymphs are phloem feeders, we concluded that the factor responsible for hampered development and elevated resistance observed in LA1840 indeed must be a mobile, vasculature‐based factor.

### The resistance phenotype can be linked to riboflavin

**Figure 4:**
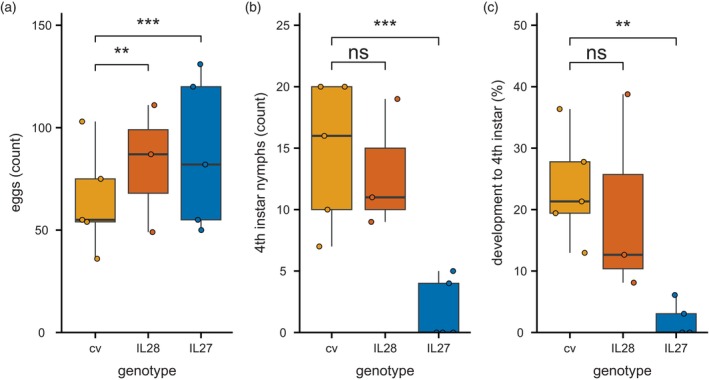
Whitefly resistance phenotype in an introgression line of *S. chmielewskii* LA1840 in a background of tomato cultivar *S. lycopersicum* cv Moneyberg. (a) Whitefly oviposition after 1 day on tomato cultivar *S. lycopersicum* Moneyberg (cv; orange), introgression line 28 (IL28; red) and introgression line 27 (IL27; blue; GLM: d.f. = 12, AIC = 212.77). (b) Total number of nymphs per clip cage developed to the fourth instar stage after 34 days on cv, IL28, and IL27 (GLM: df = 12, AIC = 79.09). (c) Development from egg to the fourth instar (%) after 34 days on cv, IL28, and IL27 [Kruskal–Wallis: *χ* ^2^(2) = 8.89, *P* = 0.01]. Boxes represent the interquartile range (IQR), displaying the median as a bold line (cv and IL27: *n* = 5, IL28: *n* = 3), whiskers 1.5 × IQR. Colored dots represent individual datapoints; black dots are outliers. The means of IL27 and IL28 were compared with those of cv using a generalized linear model or a Kruskal–Wallis rank sum test followed by a non‐parametric multiple comparison for relative effects, with *P*‐values indicated with asterisks (***P < 0.001; **P < 0.01; ns: *P* > 0.05).

Next, we aimed to identify the causal factor through linking the resistance phenotype to metabolic features obtained using an untargeted metabolomics approach. Since cv and LA1840 can be expected to produce a wide variety of different metabolites, comparing only these two accessions is likely to result in a candidate list containing many differential but unlinked metabolites. Therefore, we used a collection of ILs, where all lines contain particular regions of the LA1840 genome in a cv background (Moneyberg, a close relative of Moneymaker). A first visual scoring screen resulted in five potential resistant ILs (scores “‐” and “‐‐”; Table S2). All five potentially resistant ILs were used in a bioassay to determine the number of eggs and development to fourth instar nymphs after 34 days. In this bioassay, only one line exhibited the resistance phenotype of LA1840 (Figure S3). We compared this line (IL27) to a line that displayed a susceptible phenotype (IL28). We validated whitefly performance comparing the number of eggs and development into the fourth instar stage in detail for the two IL and the susceptible parental control. We observed a small increase in oviposition on both ILs but confirmed that the decrease in nymphal development only occurred on IL27 (Figure 4; Table 2).

**Table 2:** Number of eggs and fourth instars, and percentage of eggs developed to fourth instars per genotype including *S. lycopersicum*, accession Moneyberg (cv; *n* = 5) and introgression lines containing *S. chmielewskii* segments: IL28 (*n* = 3) and IL27 (*n* = 5)

Genotype	Eggs	Fourth instars	Development to fourth instars
	Mean (±SE)	Mean (±SE)	Mean (±SE)
cv	64.60 (±11.41)	14.60 (±2.64)	23.57% (±3.98)
IL28	82.33 (±18.05)	13.00 (±3.06)	19.84% (±9.56)
IL27	87.60 (±16.49)	1.80 (±1.11)	1.83% (±1.22)

Values are given as mean with the standard error (SE) in parentheses.

**Figure 5:**
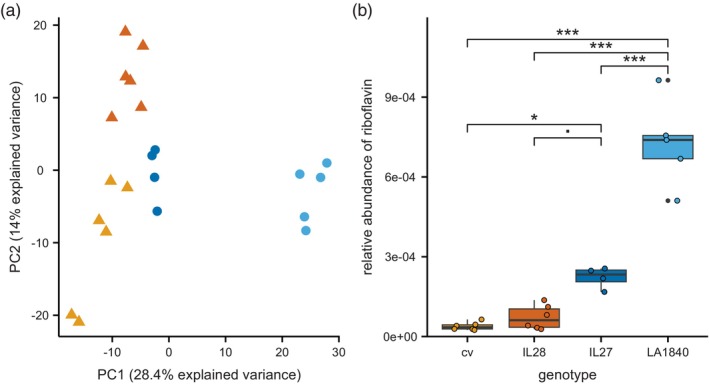
Untargeted metabolomics of leaf material. (a) Principal component analysis comparing the susceptible (triangles) tomato cultivar *S. lycopersicum* Moneyberg (cv; orange) and introgression line 28 (IL28; red) to the resistant (circles) introgression line 27 (IL27; dark blue) and wild accession *S. chmielewskii* (LA1840; light blue). (b) The relative abundance of riboflavin (the metabolite predicted to be linked to the resistance phenotype) as peak intensity normalized to the total per sample in cv, IL28, IL27, and LA1840 (anova: *F*_3,17_ = 73.59, *P* < 0.001). Boxes represent the interquartile range (IQR), displaying the median as a bold line (cv and IL28: *n* = 6; IL27: *n* = 4, LA1840: *n* = 5), whiskers 1.5 × IQR. Colored dots represent individual data points; black dots are outliers. Asterisks indicate *P*‐values from a Tukey HSD *post hoc* following an anova (***P < 0.001; *P < 0.05; ■: *P* < 0.1).

Because the ILs genetic makeup consists largely of *S. lycopersicum* cv background, with single introgressions of LA1840 DNA, we expected large similarity between their metabolic profiles and that of the cv. To identify metabolite(s) putatively underlying the resistance phenotype, we performed a UPLC‐qToF analysis on leaf material of cv, IL28, IL27, and LA1840. In total, 792 features were identified in positive ionization mode (Table S3). A principal component analysis based on these features separated LA1840 from the other genotypes along PC1, which explained 28.4% of the total variance (Figure 5a). Consistent with a small genomic introgression from LA1840, IL27 could be separated from cv and IL28 along the same PC, although to a lesser extent. This implies that the metabolic pattern of IL27 was indeed in general resembling that of IL28 and cv, except for a set of metabolites likely governed by the genomic region linked to the resistance phenotype.

To link specific metabolic features to the resistance phenotype, we applied a Random Forest (RF) machine learning approach. The genotypes were labeled “resistant” (LA1840 and IL27) or “susceptible” (cv and IL28) and a classifier model was built relying on the Gini measure for class separation. The RF model, used to compute the importance of the 792 metabolites to the resistance phenotype, had an average accuracy score of 90% ± 16% (10‐fold cross‐validation) and was significant (*P* < 0.01 based on 100 random permutations). The model identified 41 metabolic features as important and significant for class separation (*P* < 0.01 based on 100 random permutations). Out of these, 10 features exhibited a higher relative abundance (peak intensity normalized to total per sample) in the resistant plants, compared with the susceptible plants (Table S4). The relative abundance of the top candidate feature was 21‐fold higher in LA1840 compared with cv and sevenfold higher in IL27 compared with cv and was identified to be riboflavin (m/z 377.1454 [M + H]^+^; Figure 5b). The abundance of riboflavin in the susceptible IL28 was comparable to the cv. In short, we predicted the whitefly resistance phenotype to be linked to an increased relative abundance of riboflavin in leaves.

**Figure 6:**
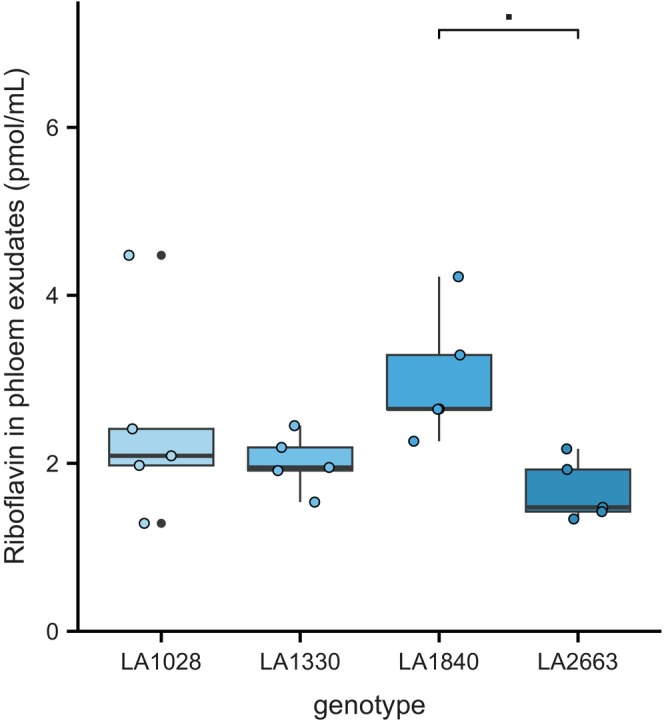
Riboflavin concentrations (pmol/mL) in phloem extracts of four *S. chmielewskii* accessions LA1028, LA1330, LA1840, and LA2663 (anova: *F*_3,16_ = 2.95, *P* = 0.06). Boxes represent the interquartile range (IQR), displaying the median as a bold line (*n* = 5), whiskers 1.5 × IQR. Colored dots represent individual datapoints; black dots are outliers. ■: Above brackets indicate *P*‐values <0.1, as obtained from a Tukey HSD *post hoc* following an anova.

To investigate whether riboflavin could also be found in the phloem of *S. chmielewskii*, we analyzed phloem exudate from the four *S. chmielewskii* accessions, collected after the initial bioassay (Figure 6; Table 3). Riboflavin was present in the phloem, especially in resistant LA1840, though overall only in very low concentration.

**Table 3:** Riboflavin concentration in phloem exudates in four *S. chmielewskii* accessions (pmol ml^−1^; *n* = 5)

Genotype	Phloem (pmol ml^−1^)
	Mean (±SE)
LA1028	2.45 (±0.54)
LA1330	2.01 (±0.15)
LA1840	3.01 (±0.34)
LA2663	1.67 (±0.16)

Values are given as mean with the standard error (SE) in parentheses.

### Altering the riboflavin level affects whitefly resistance

To validate the role of riboflavin in the resistance phenotype, we performed whitefly developmental bioassays on cv and LA1840 leaves in a hydroponics solution spiked with either riboflavin for the cv leaves or a riboflavin synthase inhibitor for the LA1840 leaves. The riboflavin inhibitor used here is a competitive inhibitor that does not completely inhibit riboflavin synthesis (Zhao et al., 2009). Targeted analysis of riboflavin in these experiments indeed confirmed the effect of the treatments on the riboflavin levels in the plants (Figure S4).

**Figure 7:**
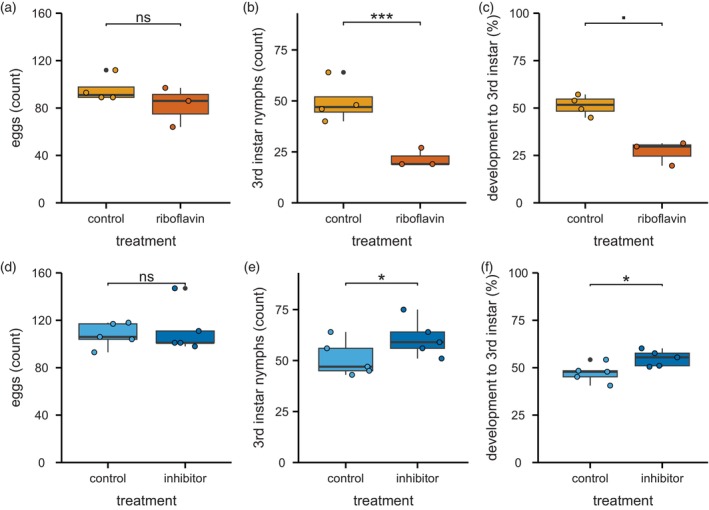
Effect of modulated riboflavin levels on whitefly resistance by riboflavin feeding to *S. lycopersicum* or chemical inhibition of riboflavin synthase in *S. chmielewskii*. (a) Whitefly oviposition after 2 days on tomato cultivar *S. lycopersicum* Moneymaker (cv) on control hydroponics solution (orange) compared with cv on hydroponics solution amended with riboflavin (riboflavin; red; 15 mg L^−1^; GLM: df = 6, AIC = 59.02). (b) Total number of nymphs per clip cage developing to the third instar stage after 17 days on control compared with riboflavin‐treated cv (GLM: df = 6, AIC = 69.67). (c) Development from egg to the third instar (%) after 17 days on control compared with riboflavin‐treated cv (Wilcoxon: *W* = 12, *P* = 0.06). (d) Whitefly oviposition after 2 days on wild accession *S. chmielewskii* (LA1840) on control hydroponics solution (light blue) compared with LA1840 on hydroponics solution amended with a riboflavin synthase inhibitor (inhibitor; dark blue; 5 mg L^−1^; GLM: df = 9, AIC = 87.29). (e) Total number of nymphs per clip cage developing to the third instar stage after 17 days on control compared with inhibitor‐treated LA1840 (GLM: df = 9, AIC = 73.81). (f) Development from egg to the third instar (%) after 17 days on control compared with inhibitor‐treated LA1840 (Wilcoxon: *W* = 2, *P* = 0.03). Boxes represent the interquartile range (IQR), displaying the median as a bold line (LA1840 control and inhibitor: *n* = 5; cv control: *n* = 4; cv riboflavin: *n* = 3), whiskers 1.5 × IQR. Colored dots represent individual datapoints; black dots are outliers. The means were compared using a generalized linear model or a Wilcoxon rank sum test, as indicated with asterisks (***P < 0.001; *P < 0.05; ■ *P* < 0.1; ns: *P* > 0.05).

When the riboflavin concentration in a cultivar leaf was increased by the addition of riboflavin to the nutrient medium, the whitefly nymphal development was hampered on that leaf, a resistance phenotype mimicking that of LA1840 (Figure 7a–c; Table 4). Although the oviposition was similar on control and riboflavin‐treated cv, the number of third instar nymphs on the riboflavin‐treated leaves was half that of the number on control leaves.

**Table 4:** Number of eggs and third instars, and percentage of eggs developed to third instars for *S. lycopersium* cv Moneymaker (cv; control: *n* = 4; riboflavin: *n* = 3) treated with riboflavin (15 mg L^−1^) and *S. chmielewskii* LA1840 (*n* = 5) treated with a riboflavin synthase inhibitor (5 mg L^−1^)

Treatment	Eggs	Third instars	Development to third instars
	Mean (±SE)	Mean (±SE)	Mean (±SE)
Genotype: cv			
Control	95.75 (±5.50)	49.50 (±5.12)	51.37% (±2.66)
Riboflavin	82.33 (±9.70)	21.67 (±2.67)	26.89% (±3.68)
Genotype: LA1840			
Control	107.60 (±4.61)	51.00 (±3.94)	47.25% (±2.23)
Inhibitor	111.60 (±9.12)	61.00 (±4.09)	54.96% (±1.88)

Values are given as mean with the standard error (SE) in parentheses.

On LA1840 leaves treated with a riboflavin synthase inhibitor, we saw the opposite effect on nymphal development (Figure 7d–f; Table 4). Again, oviposition was unaltered compared with control leaves. However, the percentage of eggs that developed to third instar stage significantly increased after treatment with the riboflavin synthase inhibitor.

## DISCUSSION

### Natural variation in whitefly resistance of *S. chmielewskii*

In the current study, we identified a vasculature‐based whitefly resistance mechanism in the wild tomato species *S. chmielewskii*, specifically accession LA1840, which affects nymphal development of the pest insect *B. tabaci*. Although cultivated tomato varieties are vulnerable to infestations of these insects, several of their wild relatives possess resistance mechanisms like the toxic and repellent compounds excreted from the glandular trichomes (Bleeker et al., 2011; Lucatti et al., 2013; Rakha et al., 2017). However, some wild tomato genotypes, including *S. chmielewskii* LA1840 under study here, do not possess such a trichome‐based line of defense (Kortbeek et al., 2021). Nevertheless, instead it appears they developed a different resistance mechanism by which they interfere with whitefly nymphal development.

In a *B. tabaci* nymph developmental screen on four *S. chmielewskii* genotypes and a cultivar, we observed a decreased development on two accessions, LA1028 and LA1840 (Figure 2). The development on these accessions was not only delayed, but there were also fewer nymphs able to reach the last instar stage. Whitefly and plant performance are influenced by external factors, effects exacerbated by a relatively long phenotyping period of 4–5 weeks. This is reflected in the differences in whitefly performance on the cultivar plants between experiments and especially in the grafting experiments where cultivar scion performance can cause varying “dilution” effects. To prevent this from disproportionately affecting the different accessions, all experiments were designed in a randomized block pattern. Although the differences between experiments prevented a direct comparison of count data between experiments, the pattern of hampered development on LA1840 compared with the cultivar plants in the same experiment was consistent in all experiments. Adult performance on LA1840 was previously found unaffected (Kortbeek et al., 2021). Adults might not be affected as much as they feed much less compared with nymphs. It might also be that the affected processes that cause hampered development in the nymphal stages are not relevant in the adults. We did however observe an effect on oviposition in several experiments, which could point to hampered adult performance (Figure 1; Table 1). However, this effect was not consistent and not observed in the riboflavin feeding assay (Figure 7a,d), thus we did not follow up on this.

The reduced nymphal development on accession LA1840 was previously noticed by de Almeida et al. (2023). The authors assumed this resistance to be caused by a trichome‐based defense mechanism, because this was the case for other genotypes included in their study (de Almeida et al., 2023). However, the glandular trichome densities on LA1840 as well as the terpenes and acylsugars produced by those trichomes were previously shown to be equally low to those on cultivar leaves (Kortbeek et al., 2021). In addition, we showed that the variation in nymphal development between the four S. *chmielewskii* accessions studied here could not be attributed to leaf surface chemistry (Figure S1). This decreased nymphal development will temper the exponential population growth, characteristic of a whitefly infestation. Whiteflies have a haplodiploid sex determination system, and sex ratios in a population may be affected by many factors, including nutrient supply by endosymbionts (Liu et al., 2024; Stansly et al., 2010; Wang et al., 2020; Yao et al., 2023). Therefore, take a simplified situation where each female whitefly lays eggs of which 50 eggs will develop into females, and as based on our findings (Table 1), 38.7% of those eggs will develop to adults on a cultivar and 9.09% on LA1840. After one generation, the population on the cultivar is over four times larger than that on LA1840, but after 10 generations, the difference in population size on the two genotypes would become 1.9 million‐fold. Thus, what seems to be a small difference in an experiment would have a much larger impact in the field.

### The resistance mechanism can be transferred from rootstocks and is likely based in the phloem

By grafting a susceptible cultivar scion on an LA1840 rootstock, we demonstrated that the resistance phenotype can be transferred to distant plant parts (Figure 3). This supports our hypothesis that the resistance observed in this wild tomato species must be caused by a vasculature‐mobile factor. We used rootstocks with multiple true leaves still attached to allow for compounds produced both in the root and shoot to be transported into the cultivar scion. Although this allowed the rootstock to continue growing its shoot, the scion too grew during the experiment. This likely caused the phloem produced in the rootstock to be diluted by the phloem from the scion, and thereby a dilution of the resistance.

Beside the formation of a phloem connection over the graft junction, the xylem connections will also be established. Although we cannot exclude a mobile resistance factor to originate from the xylem, we assume this particular resistance mechanism to be phloem‐based. Not only because whiteflies feed predominantly from the phloem and only occasionally probe the xylem vessel (Milenovic et al., 2019; Pollard, 1955), the phloem has also been shown to be the interaction site of whitefly salivary effectors and the plant‐protein targets (Naalden et al., 2024). However, contrary to the textbook models, the phloem and xylem are not two strictly separated systems, but regularly interact (Aubry et al., 2019; van Bel, 1990). Also, the phloem does not only flow from leaves to roots but is multidirectional instead.

### Linking riboflavin to impeded nymph development

To analyze the phenotype in more detail, we studied an introgression library of LA1840 made in a cultivar background. A variety of specialized metabolites was previously identified in the fruit of this IL population (Ballester et al., 2016). That study, however, focused solely on fruit phenotypes. Here, we identified one IL exhibiting the same, strong resistance phenotype of LA1840 and additionally selected a line with a susceptible phenotype (Figure 4). The large similarity between these two lines was reflected in their metabolic profiles, making them a suitable tool to help select metabolites linked to the resistance phenotype (Figure 5a).

To make this selection, we used a machine learning approach based on a RF model that was previously successfully used in our lab to link terpenes and acylsugars from glandular trichomes of a panel of wild tomato species to thrips and whitefly resistance (Kortbeek et al., 2021). Tree‐based machine learning algorithms have gained in popularity and are especially suitable for the analysis of large untargeted metabolomics datasets and the subsequent feature selection (Liebal et al., 2020). With this approach, we identified riboflavin, also known as vitamin B2, as the top candidate metabolite associated with the resistance phenotype (Figure 5b). Riboflavin is not only an essential nutrient for humans but, as the precursor of the coenzymes flavin mononucleotide and flavin adenine dinucleotide, riboflavin is also essential for many vital processes in plants (Eggers et al., 2021; Schall et al., 2020). We proceeded to validate the role of riboflavin in the resistance phenotype using nymphal performance assays on cv and LA1840 leaves in which we manipulated the riboflavin content (Figure 7). Considering that the differences in nymph development observed between grafts with cv and LA1840 rootstocks increased with each developmental stage, the effect of the treatment on the phenotype probably would have been stronger had we been able to continue this assay to fourth instar stage. This was not possible, however, due to a limited supply of the required chemicals.

### Identification of a novel resistance mechanism against whiteflies in wild tomato

The link between riboflavin and the reduced nymphal development is not so obvious, as riboflavin is an essential nutrient for the insect that plays a role in many processes, including fertility (Ju et al., 2020; Wang et al., 2020). Whiteflies are even shown to depend on riboflavin‐synthesizing endosymbionts (Wang et al., 2020). We can therefore not exclude that riboflavin is indirectly involved in the resistance phenotype, rather than directly affecting nymph development.

Such indirect involvement could potentially relate to the mechanism of riboflavin‐induced disease resistance (Dong & Beer, 2000). In their study, the application of riboflavin to *Arabidopsis thaliana* and tobacco leaves was found to induce resistance to multiple pathogens through the induction of *pathogenesis‐related* (*PR*) gene expression in a salicylic acid‐independent manner. Dong and Beer therefore proposed that riboflavin‐induced resistance is part of a novel signal transduction pathway. Since then, efforts have been made to elucidate this pathway, but the exact mechanism remains to be elucidated. Although there is consensus about ROS induction by riboflavin, the downstream induced *PR1* expression and immune response modulation (Azami‐Sardooei et al., 2010; Boubakri et al., 2013; Nie & Xu, 2016; Taheri & Tarighi, 2010; Zhang et al., 2009), other downstream steps of the signaling pathway are more controversial. Most likely, MAPK cascades are involved in the signal transduction between the ROS burst and the induction of *PR1* (Nie & Xu, 2016) and some evidence points at the involvement of JA in the pathway (Boubakri et al., 2013; Taheri & Tarighi, 2010). Other research shows that riboflavin‐induced resistance is independent of the classical defense hormones JA, SA, ABA, and ethylene (Zhang et al., 2009). Similarly, whitefly infestation causes upregulation of *PR1* in both *A. thaliana* and tomato, and as the *PR* gene regulation could not be explained by a single defense hormone, it was suggested that a novel signal transduction pathway might be involved in this defense mechanism (Puthoff et al., 2010; Zarate et al., 2007). We propose that this defense mechanism and the riboflavin‐induced resistance might be part of the same signal transduction pathway.

It has been suggested that glycosylated riboflavin might be a signal‐storage form, to be used in this alternative signal transduction pathway (Dong & Beer, 2000). Riboflavin might also be glycosylated for phloem transport and would in that form remain undetected in our MRM method. Pure riboflavin is a reactive chemical that can cause oxidative stress leading to DNA‐ and cell damage (Bergwik & Åkerström, 2020; Liang et al., 2013). Many compounds in the phloem are glycosylated (Givovich et al., 1994; Jyske et al., 2015; Knox et al., 2018; Nour‐Eldin et al., 2012; Treutter et al., 1985) and this would increase the solubility and decrease the reactivity of riboflavin in the phloem. In that case, a riboflavin‐induced signal transduction pathway discussed above might inadvertently get activated through the local deglycosylation of riboflavin at the feeding site by enzymes in the whitefly saliva, needed for the detoxification of other compounds (Huang et al., 2021), or by plant defense‐related enzymes.

Alternatively, riboflavin might directly affect nymphal development above a certain concentration. Previous research showed that when aphids (*Acyrthosiphon pisum*) harboring riboflavin‐synthesizing endosymbionts were reared on a riboflavin‐rich artificial diet, their bodyweight decreased slightly but significantly, and the development to adult stage was delayed by 1 day compared with aphids reared on a riboflavin‐free diet (Nakabachi & Ishikawa, 1999). Riboflavin might have such a direct effect through ROS production upon photodegradation of ingested riboflavin. This photosensitivity has made riboflavin a popular research topic for antimicrobial treatments, as reviewed by Farah et al. (2022). Furthermore, the photodegradation of dietary riboflavin was found to cause mortality in mosquito larvae (Lima et al., 2022). Mosquito larvae fed on a riboflavin‐rich diet exhibited a normal survival when kept in the dark. However, when the larvae were exposed to light after feeding, there was a stark increase in mortality based on the dietary riboflavin concentration (Lima et al., 2022). When considering the translucent whitefly nymphs (Figure 1) in contrast to the opaquer adults that are further shaded by their wings folded over their bodies, the photodegradation of dietary riboflavin might also play a role in this specifically nymphal resistance mechanism.

In conclusion, we make a case that a vasculature‐based mobile factor in the wild tomato accession *Solanum chmielewskii* LA1840 causes *Bemisia tabaci* nymphs feeding from this host to display a hampered development compared with their performance on a susceptible *Solanum lycopersicum* cultivar. This work highlights the importance of the phloem composition in the plant–phloem feeder interactions, which could be applied in breeding for phloem feeder resistance in tomato and other crops.

## MATERIALS AND METHODS

### Plants and insects

Plants were grown from seed in general potting soil (Jongkind Substrates) under controlled greenhouse conditions (16/8 h light/dark; 22–25°C). A new batch of plants was grown for each experiment, unless otherwise indicated, and distributed over the greenhouse compartment in a randomized block pattern.

Whiteflies were collected from a *B. tabaci* (MEAM1) population reared on cucumber plants in a climate cabinet (Snijders, Tilburg; 16/8 h light/dark; at 27.4°C) and sedated on ice for selection. Insects used in bioassays were healthy female adults that were selected using a stereomicroscope and based on the presence of a rounded, light‐yellow abdomen without discolorations. After oviposition, the adults were anesthetized with CO_2_ and carefully removed. During bioassays, nymphs in all four nymphal stages (Figure 1) were separately counted on both abaxial and adaxial sides of the leaves with a stereomicroscope while keeping the leaf attached to the plant.

### Bioassays with *S. chmielewskii* accessions

A no‐choice bioassay was performed on four *S. chmielewskii* genotypes (LA1028, LA1330, LA1840 and LA2663) and *S. lycopersicum* cv Moneymaker (cv; *n* = 5). A clip cage was attached to the fourth leaf from the top of each plant containing 10 healthy female adult whiteflies. After 72 h, the adults were removed, after which the eggs were counted and the clip cages reattached. Over the course of 24 days, the nymphs in the clip cages were recorded per developmental stage.

After the bioassay, phloem exudates and leaf material were collected from all plants from leaflets from the fourth and fifth fully expanded leaves from the apex of the shoot. An EDTA‐mediated method was used for phloem exudate collection. Per plant, five leaflets were cut off at the petiole, recut in Phloem Buffer (5 mm phosphate buffer, 5 mm EDTA) and placed in stained glass bottles with Phloem Buffer in a humidity chamber. After approximately 30 min, the petioles were washed in fresh Phloem Buffer and transferred to clean stained glass bottles with 5 ml Phloem Buffer. The leaflets were kept in the humidity chamber overnight to collect the phloem exudates in Phloem Buffer. The next morning, the phloem exudates and leaf material from the leaflets opposite to those used for phloem exudate collection were snap frozen in liquid NO_2_ and stored at −80°C until further use.

### Bioassay on grafts

Three‐ to four‐week‐old *S. lycopersicum* cv Moneymaker (cv) plants were grafted onto a rootstock with one or two true leaves of cv (cv grafts) or LA1840 (LA1840 grafts) and fixed with silicone grafting clips until fully healed. Four weeks after grafting, a clip cage was attached to one of the first leaflets from the tip of the fourth leaf from the top of the scion, with 10 adult females per clip cage (day 0; *n* = 10). The adults were removed from the clip cage after 48 h (day 2) and the eggs were counted. Starting at day 7, the nymphs in the clip cages were counted every other day for 4 weeks. Nymphs reaching the late fourth instar stage were removed from the leaflet to prevent oviposition by newly emerged adults.

### IL screen

An introgression library of LA1840 in a background of *S. lycopersicum* cv Moneyberg (cv) previously used by Ballester et al. (2016) was screened on whitefly resistance. Clip cages with 15 adult whiteflies were attached to one leaflet per plant for 24 h, after which the adults were removed. To select interesting lines, the number of fourth instar nymphs was assessed after 34 days with a visual screen with scores ranging from ++ (many fourth instar nymphs) to −− (very few fourth instar nymphs). The bioassay was then repeated with ILs of interest in which case leaflets were photographed after removal of the adults and again 34 days after the start of the bioassays. Per plant, the eggs on the first photograph were counted and compared with the number of fourth instar nymphs and exuviae on the second photograph. This was repeated with the IL that showed resistance (IL27), a susceptible IL with a largely overlapping genomic introgression (IL28) and the two parental genotypes.

### Metabolomics ILs

Leaves of the ILs of interest and the parental lines were cut off, flash‐frozen in liquid nitrogen, and homogenized (*n* = 6). Per sample, 100 g of ground plant material were extracted in 300 μl methanol containing 0.01% formic acid. The samples were briefly vortexed, subjected to sonication in ice‐cold water (20 min) and centrifuged (14 000 ***g***; 30 min). Supernatants were transferred to LC–MS vials and analyzed in positive mode on UPLC‐ESI‐qTOF according to Balcke et al. (2017, 2024). Due to the already established method for analysis in positive ionization mode, the analysis was focused on this ionization mode.

### Targeted riboflavin analysis

Phloem exudates and leaf material from the four *S. chmielewskii* genotypes collected after the initial bioassay were used for targeted analysis of riboflavin. Per sample, 10 leaf punches (20 ± 1 mg fresh weight total) were ground with metal beads after which 1 ml of 60% acetonitrile in water was added. Of the phloem exudates, a 500 ml sample was mixed with 750 ml of 60% acetonitrile in water. Both leaf and phloem samples were placed at −20°C for 30 min and centrifuged (4°C; 30 min, 10 000 ***g***). The supernatants were dried overnight in a cooled speedvac (CentriVap SpeedVac; Labconco) and resuspended in 100 μl of 10% ACN with 0.1% formic acid and 0.1% ammonium acetate in water. To all samples, 10 pm of (−)‐Riboflavin‐^13^C_4_, ^15^N_2_ (solution; Supelco) was added as an internal standard. The samples were centrifuged (4°C; 30 min) and the top 50 ml was transferred to an LC–MS vial.

The extracted samples were analyzed with an Agilent 1290 Infinity II UHPLC (www.agilent.com) coupled to an Agilent 6470 LC/TQ (gas: 340°C, 10 L min^−1^; sheath gas: 400°C, 11 L min^−1^) and equipped with an ESI + Agilent Jet Stream ion source (35 psi). Of each sample, 1 μl was injected and separated on an InfinityLab Poroshell 120 PFP column (2.1 × 100 mm, 1.9 μm; 35°C; 0.35 ml min^−1^). The mobile phase consisted of A (10 mm ammonium acetate, 0.1% formic acid in water) and B (99% acetonitrile, 0.1% formic acid in water) in an A:B gradient of 90:10 at 0–1.20 min, 5:95 at 5.0–7.75 min, 80:20 at 8.0 min to end of run. The molecules were ionized in positive mode and detected in MRM mode as m/z 377.1 > 243.1, m/z 377.1 > 198.1, and m/z 377.1 > 172.1 for riboflavin and as m/z 383.0 > 249.0 for the internal standard. Riboflavin and internal standard peaks were quantified using MassHunter software (Agilent). All solvents were LC–MS grade from Biosolvent.

### Hydroponics assays

The fourth leaf from the top was taken from *S. lycopersicum* cv Moneyberg (cv) and LA1840 plants and placed in 50 ml tubes wrapped with aluminum foil, with a hydroponics solution of 1:1:1:1000 FloraGrow:FloraBloom:FloraMicro:water (General Hydroponics). The tubes were closed with only a small hole in the lid for the petiole to prevent evaporation of the solution. The cv leaves were control or riboflavin‐treated (*n* = 5), where riboflavin‐treated plants had an increasing concentration of riboflavin added to the hydroponics solution starting at 5 mg L^−1^ and increasing by 5 mg L^−1^ twice a week with every renewal of the solutions to a final concentration of 20 mg L^−1^. This gradual increase in riboflavin concentration was based on pilot experiments, in which a rapid increase in riboflavin concentration was found to cause toxicity to the plant. The LA1840 leaves were control or inhibitor‐treated (*n* = 5). For the inhibitor‐treated leaves, 5 mg L^−1^ was added of the riboflavin synthase inhibitor 3‐(4‐chlorophenyl)‐1‐(3‐methylbenzoyl)‐5‐(trifluoromethyl)‐4,5‐dihydro‐1H‐pyrazol‐5‐ol (ChemDiv ID: 3852‐0429).

A clip cage was attached to each leaf to which 10 adult whiteflies were added. After 48 h, the adults were removed, and the eggs were counted. After the first eggs had hatched, all nymphs were counted twice a week. Due to the setup in hydroponics, cuttings could not be kept long enough for whitefly nymphs to reach fourth instar stage, which takes about 5 weeks in total. We therefore used third instar stage as the end point here. After the bioassay, all leaves were collected, and their riboflavin levels were analyzed with the above‐mentioned method.

### Volatile analysis

Volatile extraction and analysis were adapted from Kortbeek et al. (2021). In short, volatiles were extracted from five plants per genotype, two leaflets per plant as technical replicates. Leaflets were weighed and quickly (< 10 sec) washed by pipetting 500 μl *n*‐hexane spiked with 0.5 ng μl^−1^ benzyl acetate as an internal standard over it five times. The extracts were dried with 10 mg Na_2_SO_4_ (s) and centrifuged (5 min, 10 000 ***g***), and the supernatant was stored in glass vials under N_2_ (g) at −20°C until analysis.

Volatile analysis was done using an Agilent 7890A gas chromatograph coupled to a 7200 accurate mass time‐of‐flight (TOF) mass spectrometer, as described in Kortbeek et al. (2021). Terpenes of interest were verified by analytical standards. Base‐peak ions of metabolites were used for peak integration, and peak areas were corrected by the internal standard and sample dilution and normalized for leaf fresh weight.

### Data analysis

All data analyses are performed in R (v4.1.1; R Core Team, 2022) using Rstudio (v2022.02.3+492; RStudio Team, 2018) and figures were made with ggplot2 (v3.3.6; Wickham, 2016) and patchwork (v1.1.1; Pedersen, 2023) packages. Normality and homogeneity of variance were tested with a Shapiro–Wilk test and a Levene's test, respectively. If assumptions of normality and homogeneity of variance were met, a parametric test was used; otherwise, a nonparametric equivalent was chosen. Percentage data was compared with a Wilcoxon rank sum test for comparison of two groups, while percentage data for three or more groups was compared using a Kruskal–Wallis rank sum test. The Kruskal–Wallis rank sum test was followed by a non‐parametric multiple comparison for relative effects as *post hoc*, using the nparcomp package (Konietschke et al., 2015).

For the graft bioassay, the cumulative percentage of nymph development to the fourth instar stage over time was used to fit a three‐parameter log‐logistic model per graft type using the DRC package (v3.0‐1; Ritz et al., 2015). The parameters of the model describe the maximum of the curve, the slope of the linear section of the curve, and the 50% emergence time. The area under the curve for the nymphal development was calculated for the proportional data using the DescTools package (v0.99.45; Signorell, 2023).

### Random Forest

Metabolic features and their standardized peak areas were used as the input matrix X while plants from the different genotypes (IL27, IL28, LA1840 and cv) were labeled with their phenotypes (resistant: LA1840 and IL27; sensitive: cv and IL28) to obtain classes. The ranger package (v0.13.1; Wright & Ziegler, 2017) was used to perform a RF classification analysis (10‐fold cross‐validation; train/test data ratio: 0.70; 10.000 trees; minimal node size: 3). The permutation method (*N* = 100 permutations) was used to calculate the significance of both model and features.

## AUTHOR CONTRIBUTIONS

RCS, MdV, and PMB planned and designed the research. L‐AMD and AvD performed experiments. L‐AMD, AvD, GB, and MG analyzed the data. L‐AMD and MG made the figs. L‐AMD, AvD, MG, and PMB wrote the manuscript. All authors read and approved the manuscript.

## CONFLICT OF INTEREST

The authors declare that there are no conflicts of interest.

## PERMISSION TO REPRODUCE MATERIAL FROM OTHER SOURCES

All material is original.

## Supporting information


**Figure S1.** Terpenes from volatile extractions of *Solanum lycopersicum* cv Moneymaker (MM) and Moneyberg (MB) and four *Solanum chmielewskii* accessions.
**Figure S2.** Whitefly oviposition and hatching on *Solanum lycopersicum* cv Moneymaker scions grafted on *Solanum chmielewskii* LA1840 rootstocks.
**Figure S3.** Nymph developmental bioassay screen on introgression lines with parental lines *Solanum lycopersicum* cv Moneyberg (cv) and *Solanum chmielewskii* LA1840.
**Figure S4.** Riboflavin concentrations after hydroponics experiment.
**Table S1.** Number of eggs and hatched eggs, and hatching rate as percentage on grafts.
**Table S2.** Resistance screen of introgression lines.
**Table S3.** Full list of metabolites from untargeted metabolomics.
**Table S4.** Candidate metabolites from untargeted metabolomics.

## Data Availability

All supporting data and materials can be accessed; the source code can be found at https://github.com/BleekerLab/Riboflavin/ (release v1.0.0).
