# Supplementary material for: A phloem‐based defense mechanism linked to elevated riboflavin levels in wild tomato Solanum chmielewskii impedes whitefly nymphal development
Source: Plant J. 2025 Jul 25;123(2):e70363. doi: 10.1111/tpj.70363 (PMC12296259; doi:10.1111/tpj.70363)
Supplement: Supplementary file 1 — Figure S1. Terpenes from volatile extractions of Solanum lycopersicum cv Moneymaker (MM) and Moneyberg (MB) and four Solanum chmielewskii accessions. Figure S2. Whitefly oviposition and hatching on Solanum lycopersicum cv Moneymaker scions grafted on Solanum chmielewskii LA1840 rootstocks. Figure S3. Nymph developmental bioassay screen on introgression lines with parental lines Solanum lycopersicum cv Moneyberg (cv) and Solanum chmielewskii LA1840. Figure S4. Riboflavin concentrations after hydroponics experiment. Table S1. Number of eggs and hatched eggs, and hatching rate as percentage on grafts. Table S2. Resistance screen of introgression lines. Table S3. Full list of metabolites from untargeted metabolomics. Table S4. Candidate metabolites from untargeted metabolomics. [file TPJ-123-0-s001.zip › tpj70363-sup-0001-Supinfo.docx]

## Supporting Information

*
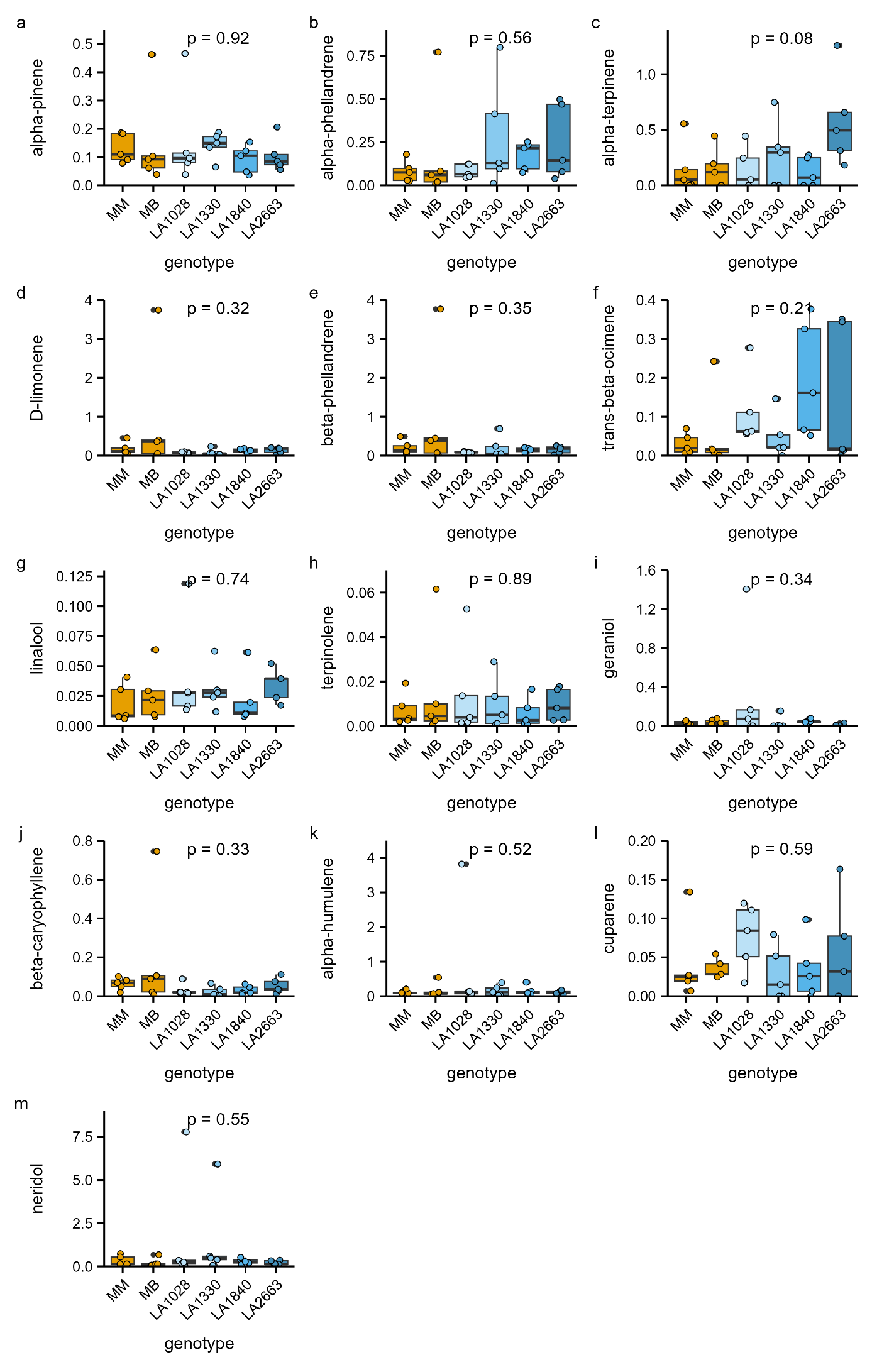
*

**Fig. S1** Terpenes from volatile extractions of *Solanum lycopersicum* cv Moneymaker (MM) and Moneyberg (MB) and four *Solanum chmielewskii* accessions. Values on the y-axis represent corrected peak areas normalised to leaf fresh weight, compared using ANOVA with p-values indicated per plot. Boxes represent the interquartile range (IQR), displaying the median as bold line and whiskers 1.5*IQR. Coloured dots represent individual datapoints (n=5).


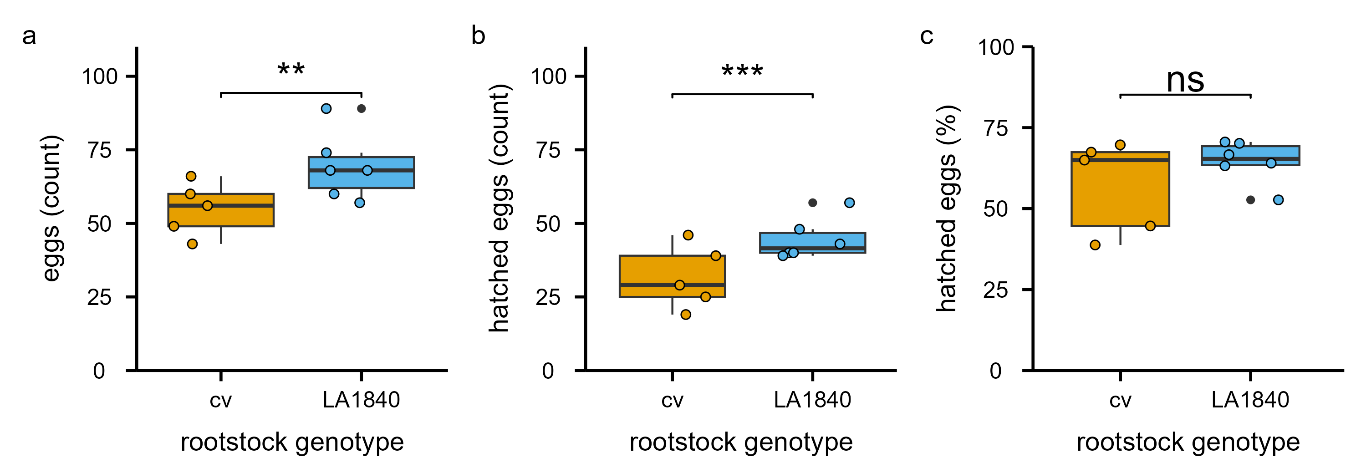


**Fig. S2** Whitefly eggs on *Solanum lycopersicum* cv Moneymaker scions grafted on *Solanum chmielewskii* LA1840 rootstocks. a: Whitefly oviposition on Moneymaker scions grafted on *S. lycopersicum* Moneymaker rootstocks (cv; orange) compared to Moneymaker scions grafted on *S. chmielewskii* LA1840 (blue; GLM: df=10, AIC=84.74). b: Total number of hatched eggs per clip-cage on cv|LA1840 grafts compared to cv|cv grafts (GLM: df=10, AIC=84.13). c: Hatching of eggs (%) on cv|LA1840 grafts compared to cv|cv grafts (Wilcoxon: W=11, p=0.53). Boxes represent the interquartile range (IQR), displaying the median as bold line (cv: n=5; LA1840: n=6), whiskers 1.5*IQR. Coloured dots represent individual datapoints, black dots are outliers. The means were compared using a Generalised Linear Model or Wilcoxon rank sum test, as indicated with asterisks (***: p<0.001; **: p<0.01; ns: p>0.05)


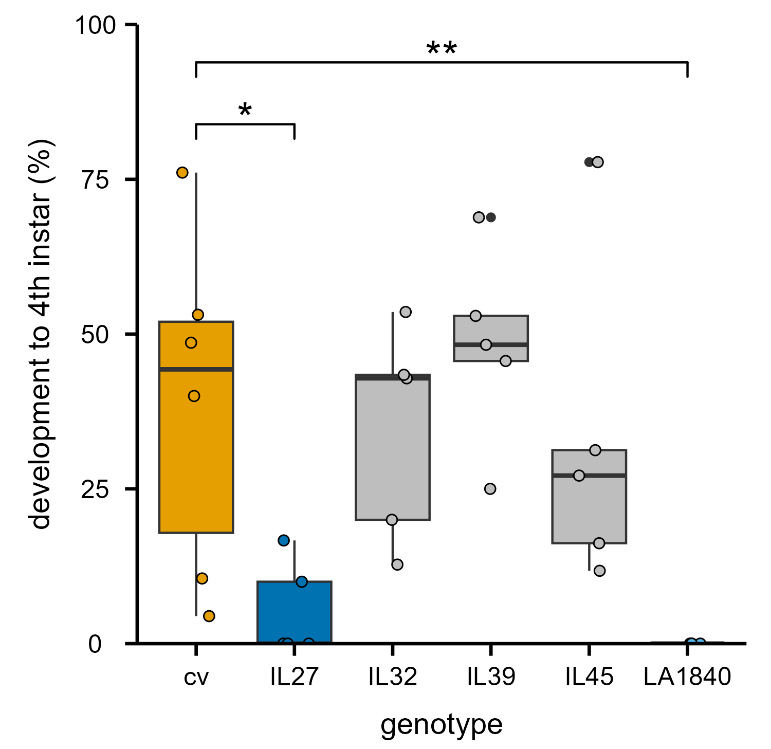


**Fig. S3** Nymph developmental bioassay screen on introgression lines with parental lines *Solanum lycopersicum* cv Moneyberg (cv) and *Solanum chmielewskii* LA1840. Follow-up on visual screen with all lines possibly showing resistance (Kruskal-Wallis: χ^2^(5)=17.41, p=0.003). All values for LA1840 were zero. Boxes represent the interquartile range (IQR), displaying the median as centre line and whiskers 1.5*IQR. Dots represent individual datapoints (cv n=6; ILs n=5; LA1840 n=4). Asterisks indicate p-values from the comparison of IL27, IL32, IL39, IL45, and LA1840 to cv using a a Kruskal-Wallis rank sum test followed by a nonparametric multiple comparison for relative effects (**: p<0.01; *: p<0.05).


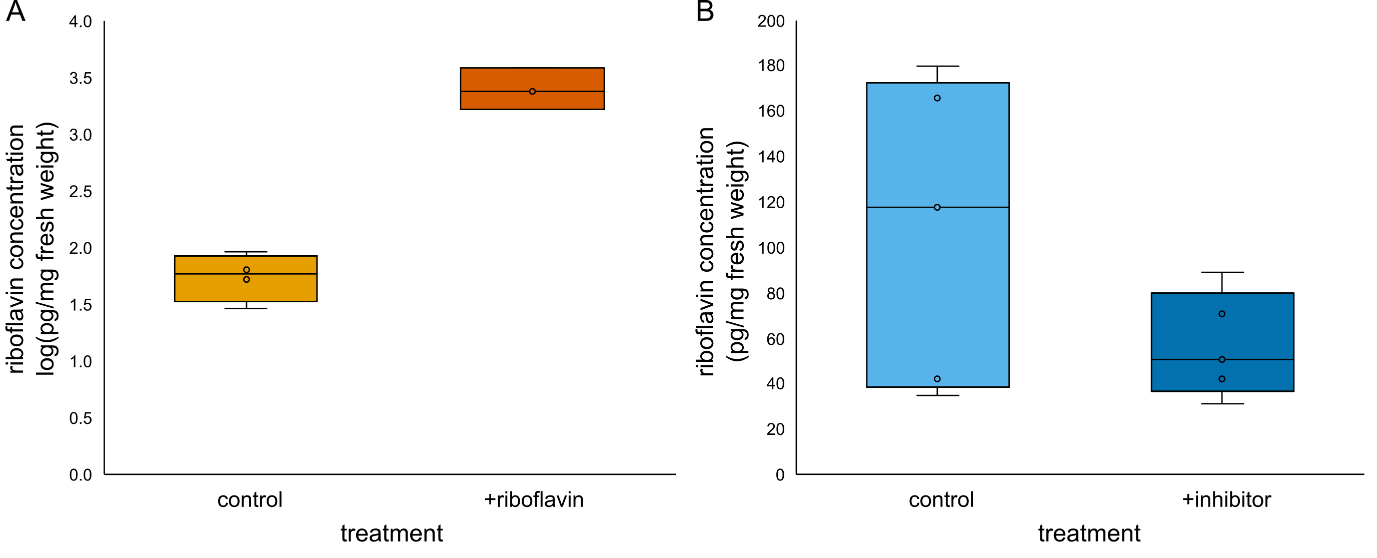


**Fig. S4** Riboflavin concentration after hydroponics experiment. a: Leaves of *Solanum lycopersicum* cv Moneymaker after control (yellow) an +riboflavin (red; 15 mg/L) treatment. b: Leaves of *Solanum chmielewskii* LA1840 after control (light blue) and +inhibitor (dark blue; 5 mg/L) treatment. Boxes represent the interquartile range (IQR), displaying the median as centre line and whiskers 1.5*IQR. Dots represent individual datapoints (cv control n=4; cv +riboflavin n=3; LA1840 n=5)

**Table S1.** Number of eggs and hatched eggs, and hatching rate as percentage per graft type cv|cv (n=5) and cv|LA1840 (n=6), as rootstock genotype

|  | **Eggs** | | | **Hatched** | | | **Hatching rate** | | |
| --- | --- | --- | --- | --- | --- | --- | --- | --- | --- |
| **Rootstock genotype** | Mean | (±SE) | Mean | | (±SE) | Mean | | (±SE) |  |
| cv | 54.80 | (±4.04) | 31.60 | | (±4.85) | 57.11% | | (±6.40) |  |
| LA1840 | 69.33 | (±4.66) | 44.50 | | (±2.84) | 64.57% | | (±2.68) |  |

**Table S2.** Resistance screen based on visual inspection of *Bemisia tabaci* nymphal development on introgression lines (IL) originating from a cross between parental lines *Solanum lycopersicum* cv Moneyberg (cv) and *Solanum chmielewskii* LA1840. Plus and minus symbols represent the number of 4th instar nymphs at end of screen (34 days), with ++ as the highest number, + as high, - as low and -- as the lowest.

| **Introgression line** | **Score** |
| --- | --- |
| IL06 | ++ |
| IL27 | - |
| IL28 | + |
| IL29 | + |
| IL30 | ++ |
| IL31 | + |
| IL32 | - |
| IL33 | + |
| IL34 | ++ |
| IL35 | + |
| IL36 | ++ |
| IL37 | ++ |
| IL38 | ++ |
| IL39 | -- |
| IL40 | ++ |
| IL41 | ++ |
| IL42 | + |
| IL43 | - |
| IL44 | +- |
| IL45 | - |
| IL46 | ++ |
| IL47 | ++ |
| IL48 | ++ |
| IL49 | + |
| IL50 | + |
| IL51 | ++ |
| IL52 | ++ |
| IL53 | ++ |
| cv | ++ |

**Table S3.** Full list of metabolic features resulting from untargeted metabolomics UPLC-qToF analysis on leaf material. Available as separate Excel file.

**Table S4.** Significant (p < 0.01) candidate metabolites identified from the analysis of untargeted metabolomics data with a Random Forest model that are more abundant in resistant genotypes (IL27 and LA1840) compared to sensitive genotypes (Moneyberg, IL28).

| **rt (min)** | **m/z [M+H]^+^** |
| --- | --- |
| 9.02001 | 377.14538 |
| 17.57283 | 1197.59505 |
| 30.52115 | 511.28713 |
| 17.36594 | 1047.56008 |
| 17.43474 | 1066.55775 |
| 17.36324 | 1046.55674 |
| 16.78701 | 624.82529 |
| 16.76277 | 624.32432 |
| 32.41230 | 540.32275 |
| 15.70570 | 1073.53700 |
